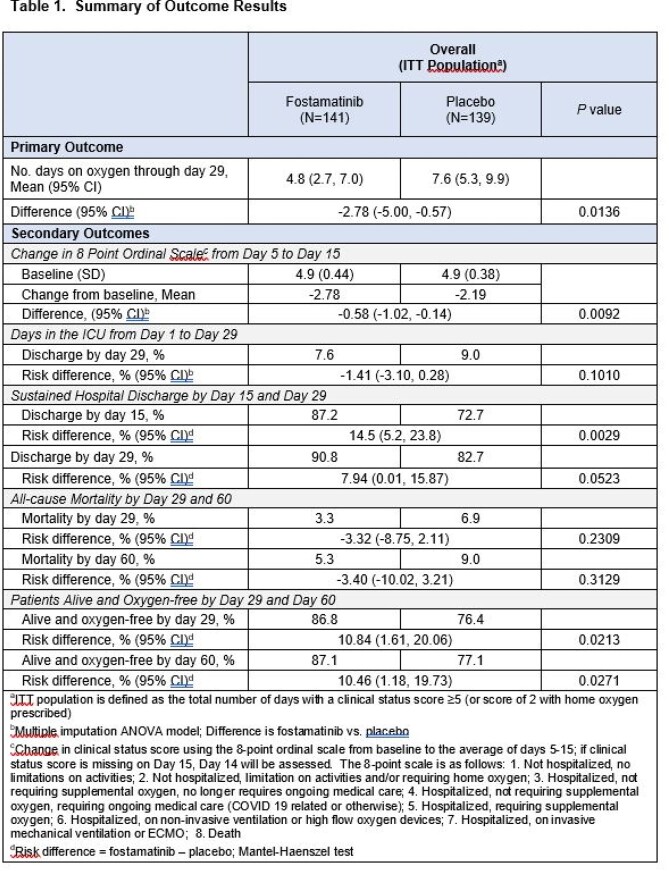# 88. Fostamatinib for the Treatment of Hospitalized Patients With COVID-19 Who Required Oxygen Supplementation: Results of a Phase 3 Trial

**DOI:** 10.1093/ofid/ofad500.004

**Published:** 2023-11-27

**Authors:** Deepa B Gotur, Anuj Malik, Vadim Markovtsov, Lucy Yan, Wolfgang Dummer, Ziad Mallat

**Affiliations:** Weill Cornell Medical College and Houston Methodist Hospital, Houston, TX; Ascension St. John's Medical Center, Tulsa, Oklahoma; Rigel Pharmaceuticals, Inc., South San Francisco, California; Rigel Pharmaceuticals, South San Francisco, California; Rigel Pharmaceuticals, South San Francisco, California; University of Cambridge, Cambridge, England, United Kingdom

## Abstract

**Background:**

Severe COVID-19 is linked to hyperactivation of the host immune response involving signaling through multiple spleen tyrosine kinase (SYK) pathways. Fostamatinib is an orally administered potent and selective inhibitor of SYK that has been approved in the US, Canada, Japan, and Europe for the treatment of chronic immune thrombocytopenia (ITP).

**Methods:**

We conducted a double-blind, randomized, placebo-controlled phase 3 trial of fostamatinib in adults hospitalized for COVID-19 who required oxygen supplementation (NCT04629703). Patients (pts) were randomly assigned 1:1 to receive fostamatinib (150 mg BID administered orally) or placebo for 14 days. All pts additionally received standard of care at their hospital. The primary endpoint was days on oxygen (days 1-29).

**Results:**

A total of 280 pts underwent randomization (with 141 assigned to fostamatinib and 139 to placebo). The primary endpoint was met; those who received fostamatinib had lower mean days on oxygen than those who received placebo (4.8 vs. 7.6 days, *P=0.0136*; **Table 1**).

Fostamatinib showed significance or trend towards significance in all secondary endpoints of reducing mortality and morbidity compared to placebo. The mean change in the 8-point ordinal score from baseline to the average of Day 5 to 15 was significantly improved in pts who received fostamatinib vs. placebo (*P=0.0092,***Table 1**). Furthermore, 6 pts were enrolled with a baseline ordinal score of 6 (3 in each group). All patients in the fostamatinib group survived, and all in the placebo group died by Day 30. A significantly higher proportion of pts who received fostamatinib were discharged from the hospital by Day 15 compared to placebo (*P=0.0029,***Table 1**). Significantly more patients were alive and oxygen-free by Day 29 and Day 60 with fostamatinib treatment in comparison to placebo (*P=0.0213* and *P=0.0271*, respectively, **Table 1**). Treatment-emergent adverse events were consistent with previous studies and were similar between the 2 groups.

**Conclusion:**

The addition of fostamatinib to standard of care treatment resulted in significantly fewer days on oxygen, an improved 8-point ordinal scale score, and significantly more patients alive and oxygen-free by Day 60 compared to placebo in pts with COVID-19 requiring hospitalization and supplemental oxygen.

**Disclosures:**

**Deepa B. Gotur, MD, FCCP, FCCM**, GSK: Advisor/Consultant|GSK: Speaker at ATS|Moderna: Advisor/Consultant **Vadim Markovtsov, PhD**, Rigel Pharmaceuticals, Inc.: Employee|Rigel Pharmaceuticals, Inc.: Stocks/Bonds **Lucy Yan, MD, PhD**, Rigel Pharmaceuticals, Inc.: Employee|Rigel Pharmaceuticals, Inc.: Stocks/Bonds **Wolfgang Dummer, MD, PhD**, Rigel Pharmaceuticals, Inc.: Stocks/Bonds.

**Figure:**